# Knockout of the non-essential gene SUGCT creates diet-linked, age-related microbiome disbalance with a diabetes-like metabolic syndrome phenotype

**DOI:** 10.1007/s00018-019-03359-z

**Published:** 2019-11-13

**Authors:** Joanna Niska-Blakie, Lakshmi Gopinathan, Kia Ngee Low, Yang Lay Kien, Christine M. F. Goh, Matias J. Caldez, Elisabeth Pfeiffenberger, Oliver S. Jones, Chee Bing Ong, Igor V. Kurochkin, Vincenzo Coppola, Lino Tessarollo, Hyungwon Choi, Yoganathan Kanagasundaram, Frank Eisenhaber, Sebastian Maurer-Stroh, Philipp Kaldis

**Affiliations:** 1 Institute of Molecular and Cell Biology (IMCB), A*STAR (Agency for Science, Technology and Research), 61 Biopolis Drive, Proteos #3-09, Singapore, 138673 Republic of Singapore; 2 Bioinformatics Institute (BII), A*STAR, Singapore, 138671 Republic of Singapore; 3 Department of Biochemistry, National University of Singapore (NUS), Singapore, 117597 Republic of Singapore; 4 Department of Medicine, National University of Singapore (NUS), Singapore, 117597 Republic of Singapore; 5 Department of Biological Sciences (DBS), National University of Singapore (NUS), 14 Science Drive 4, Singapore, 117597 Republic of Singapore; 6 Department of Cancer Biology and Genetics, The Ohio State University, 988 Biomedical Research Tower, 460 West 12th Ave, Columbus, OH 43210 USA; 7 Mouse Cancer Genetics Program, National Cancer Institute, NCI-Frederick, Bldg. 560, 1050 Boyles Street, Frederick, MD 21702-1201 USA; 8 School of Computer Science and Engineering (SCSE), Nanyang Technological University (NTU), Singapore, 637553 Republic of Singapore; 9 Department of Clinical Sciences, Lund University, Clinical Research Centre (CRC), Box 50332, 202 13 Malmö, Sweden

**Keywords:** Glutaric aciduria type 3 (GA3), C7orf10, Sugct, Gut microflora, Metabolomics, Lipids, Obesity

## Abstract

SUGCT (*C7orf10*) is a mitochondrial enzyme that synthesizes glutaryl-CoA from glutarate in tryptophan and lysine catabolism, but it has not been studied in vivo. Although mutations in *Sugct* lead to Glutaric Aciduria Type 3 disease in humans, patients remain largely asymptomatic despite high levels of glutarate in the urine. To study the disease mechanism, we generated *SugctKO* mice and uncovered imbalanced lipid and acylcarnitine metabolism in kidney in addition to changes in the gut microbiome. After *SugctKO* mice were treated with antibiotics, metabolites were comparable to WT, indicating that the microbiome affects metabolism in *SugctKO* mice. SUGCT loss of function contributes to gut microbiota dysbiosis, leading to age-dependent pathological changes in kidney, liver, and adipose tissue. This is associated with an obesity-related phenotype that is accompanied by lipid accumulation in kidney and liver, as well as “crown-like” structures in adipocytes. Furthermore, we show that the *SugctKO* kidney pathology is accelerated and exacerbated by a high-lysine diet. Our study highlights the importance of non-essential genes with no readily detectable early phenotype, but with substantial contributions to the development of age-related pathologies, which result from an interplay between genetic background, microbiome, and diet in the health of mammals.

**Electronic supplementary material:**

The online version of this article (10.1007/s00018-019-03359-z) contains supplementary material, which is available to authorized users.

## Introduction

**Fig. 1:**
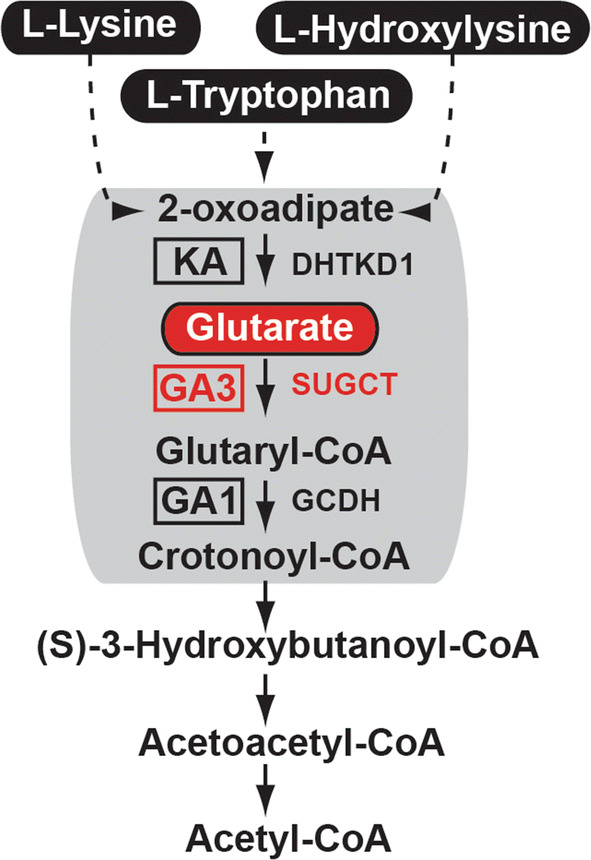
Pathway of lysine–tryptophan catabolism. Mitochondrial enzymatic defects in tryptophan, lysine, and hydroxylysine degradation pathway found in alpha-ketoadipic aciduria, GA1, and GA3 diseases. *KA* ketoadipic aciduria, *DHTKD1* dehydrogenase E1 and transketolase domain containing 1; [72]; *GCDH* glutaryl-CoA dehydrogenase, *SUGCT* succinate hydroxymethylglutarate CoA-transferase

Although mitochondrial enzymes are useful therapeutic targets to combat metabolic disorders, such as obesity and diabetes, many of them still lack proper characterization [1, 2]. *C7orf10* (*SUGCT*) is a gene with mitochondrial functions but unknown physiological role. Mutations in *Sugct* lead to a non-functional protein product that causes glutaric aciduria type 3 (GA3) disease in humans [3, 4]. Three types of glutaric aciduria diseases have been characterized [5]. Glutaric aciduria type 1 (GA1) develops due to mutations in glutaryl-CoA dehydrogenase (*GCDH*) (Fig. 1), which results in severe neurologic defects [6], while glutaric aciduria type 2 (GA2) is caused by mutation of genes involved in electron transfer in the mitochondrial respiratory chain [7]. GA3 arises from the lack of function of the *SUGCT* gene, which disrupts mitochondrial catabolism of lysine, hydroxylysine, and tryptophan (Fig. 1). *SUGCT* is a well-conserved enzyme across species and encodes a succinate hydroxymethylglutarate CoA-transferase, which converts glutarate into glutaryl-CoA [3, 4]. Although many mutations have been identified in human *SUGCT*, only a few are thought to be associated with GA3 including two nonsense mutations (Arg108Ter and Arg142Ter) and one missense arginine to tryptophan replacement (Arg336Trp), being previously characterized as Arg299Trp due to a different reference mRNA used [3]. According to the ExAC Browser [8], the frequencies of the GA3-linked Arg108Ter and Arg336Trp mutations are approximately 1/3000 and 1/200 worldwide, respectively. Despite the high frequency of *C7orf10* allele nonsense mutations, until now, only 12 patients with GA3 have been reported in the medical literature, with glutaric acid being the only significantly altered metabolite associated with the disease [3, 9–11]. In contrast to GA1 and GA2, patients with GA3 do not share distinctive symptoms, among which gastroenteritis, developmental delays, and dysmorphic features are reported, but other patients often remain asymptomatic [3, 9–11]. Therefore, unlike GA1 and GA2, the GA3 disease is poorly understood and underdiagnosed. Interestingly, a patient with GA3 disease was reported with gastrointestinal disturbances [11], and after treatment with antibiotics, the symptoms were alleviated, which could potentially suggest a role of the gut microflora in the GA3 disease.

Driven by the ambiguous symptoms in patients with GA3, we generated *Sugct* knockout mice (hereinafter referred to as *SugctKO*) to determine in vivo functions of SUGCT in mammals. We used non-targeted global metabolomic profiling of mouse kidney and plasma, which is a powerful tool for assessing biochemical metabolites in various biological contexts. Loss of *Sugct* evoked changes of the gut microbiome-dependent metabolism resulting in acylcarnitine and bacterial metabolite accumulation in kidney. These symptoms are aggravated with age or when mice are fed high-lysine diet, which resulted in an obesity-related phenotype that is accompanied by lipid accumulation in kidney and liver, as well as “crown-like” inflammatory structures in adipocytes. Our findings suggest that mutations in *Sugct* causing GA3 disease need to be studied in the context of the gut microflora, diet, and age.

## Results

### Generation of *Sugct* knockout mice

To establish the role of the *C7orf10* gene in vivo in mammals, we generated *Sugct* knockout mice by inserting *loxP* sites flanking the third exon of the *Sugct* gene (Figure S1a, Table S1). The targeting vector was electroporated into embryonic stem cells (ESCs) to generate heterozygous *Sugct*^+*/*flox^ ESCs via homologous recombination (Figure S1b). *Sugct*^+*/*flox^ ESCs were then injected into mouse blastocysts and targeted mice were obtained by standard procedures [12]. *Sugct*^flox/flox^ were bred with β-actin-cre mice to generate heterozygous null (*Sugct*^+/null^) animals, which were then intercrossed to get homozygous null animals (*Sugct*^null/null^; hereinafter referred to as *SugctKO*). PCR genotyping of *Sugct*^+*/*flox^ and *SugctKO* mice revealed bands at the expected sizes (Figure S1c). Homozygous *SugctKO* mice were obtained at the typical Mendelian frequencies (Figure S1d) and were viable.

To investigate *Sugct*/SUGCT expression levels in various tissues and verify the efficiency of the constructed *SugctKO*, we isolated RNA and proteins from several WT and *SugctKO* organs. Unlike in the mutant animals, we detected the expression of *Sugct*/SUGCT in WT kidney and liver at the mRNA and protein levels, respectively (Figure S1e, f). Both tissues are known to contain a high number of mitochondria due to their elevated metabolic rate and are often challenged by toxins and pathogens. Interestingly, it has been shown using single-cell transcriptomics in mouse kidney that *Sugct* is mainly expressed in proximal tubules, which are immune responders to toxic injuries [13]. Accumulation of urinary metabolites in kidney, where SUGCT is mostly expressed could cause deleterious phenotypes, and therefore, we started our study with investigations of renal mouse tissue.

### Metabolic changes in *SugctKO* mouse kidney

**Fig. 2:**
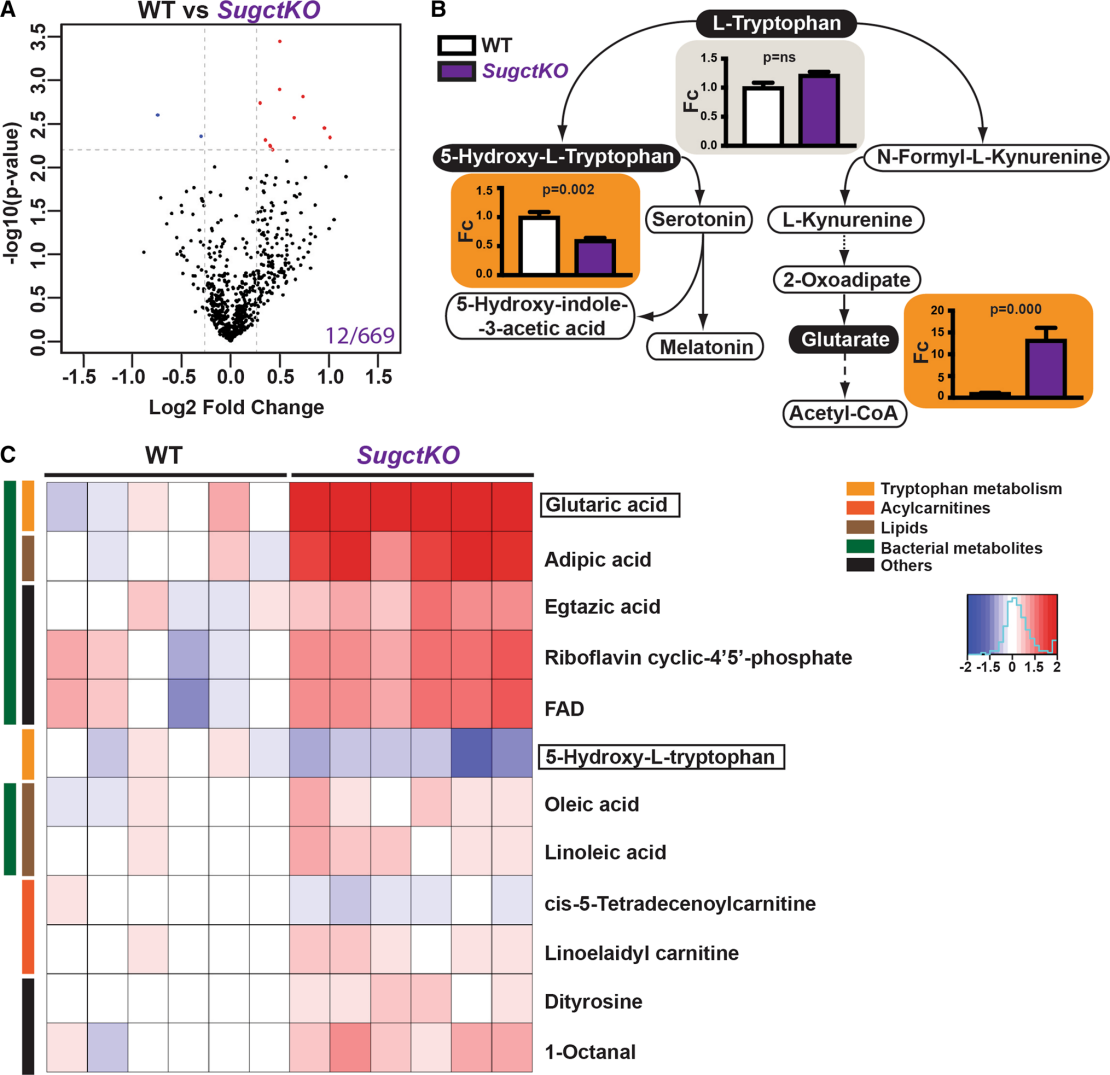
Mass-spectrometry-based metabolite profiling in *SugctKO* mouse kidney. Untargeted LC–MS was performed from WT and *SugctKO* mouse kidney using six biological and three technical replicates. **a** Volcano plot of 669 metabolites detected in *SugctKO* vs WT mouse kidney. The volcano plot was generated as a log scaled axes of fold change (Log2, *x*-axis) and *p* value (− log10, *y*-axis). Significantly altered metabolites (*q* value = 0.15, *F*_c_ ≥ 1.2) are indicated by dashed grey lines and colored in red and blue representing up- and downregulated metabolites, respectively. Note that glutarate was excluded from the Volcano plot since the scale would be vastly different. **b** l-tryptophan, 5-hydroxy-l-tryptophan, and glutarate levels in tryptophan catabolism from WT vs *SugctKO* mouse kidney. Orange and grey boxes indicate statistically significant (*q* value = 0.15, *F*_c_ ≥ 1.2) and nonsignificant metabolite levels, respectively. Statistical analysis was done using two-tailed parametric paired *t* test. **c** Heatmap depicting up- (red) and downregulated (blue) compounds (*q* value = 0.15, Fc ≥ 1.2) from WT vs *SugctKO* mouse kidney. Metabolites are clustered according to the following classes: tryptophan metabolism (yellow), acylcarnitines (orange), lipids (brown), metabolites of bacterial origin (green), and others (black)

Patients with GA3 disease are known to excrete high levels of glutarate with no significant changes of acetylcarnitine, 3-hydroxyglutarate, glutarylcarnitine, and glutarylglycine in the urine [3]. To investigate whether these metabolites can be detected in *SugctKO* mice, we performed untargeted metabolomics using Liquid Chromatography–Mass Spectrometry (LC–MS) in extracts isolated from WT and *SugctKO* kidney. All experimental mice were co-housed and matched gender- (male), age- (15 weeks), and backgroundwise (C57BL/6J), which means that usually, 2 wild type and 2 *SugctKO* mice were housed in a single cage. We detected a total of 669 compounds and tentatively annotated metabolites with an associated unique molecular mass, which in numerous cases was followed up by identity confirmation either via chemical standards or using metabolite fragmentation against libraries for possible matches [14] (Tables S2, S3, see “Materials and methods”). The data were visualized in a Volcano plot representing the differences in detected metabolites between WT and *SugctKO* mouse kidney (Fig. 2a). Among all identified compounds, differences in 12 metabolites were considered significant (2.2% of all metabolites; *q* value of 0.15, 15% False Discovery Rate (FDR), *F*_c_ ≥ 1.2). Importantly, amidst shortlisted metabolites were those involved in tryptophan catabolism (Fig. 2b). In line with the urinary metabolic alterations characteristic for patients with GA3 [3], the 11-fold increase in glutarate levels was the most prominent in *SugctKO* mouse kidney (Fig. 2b, c). Besides glutarate upregulation, we did not detect significant changes in acetylcarnitine, 3-hydroxyglutarate, glutarylcarnitine, and glutarylglycine in *SugctKO* mouse kidney (Figure S2a, Table S2) consistent with the previous clinical reports for GA3 [3]. Unfortunately, due to technical limitations, we were unable to detect lysine and its co-metabolites. Intriguingly though, tryptophan degradation via 5-hydroxy-l-tryptophan (Fig. 2b, c) was found downregulated despite unchanged tryptophan levels (Fig. 2b), which might suggest alternative degradation pathways for tryptophan. Thus, our results indicate that besides the reported altered levels of glutarate in urine of patients with GA3, there may be also changes in other metabolites.

To investigate whether we can identify other metabolites related to GA3 in *SugctKO* mice, we expanded the analysis of our metabolomic data. The vast majority of significantly altered kidney metabolites (80%) were upregulated in *SugctKO* mice with only few downregulated, suggesting that SUGCT is a “repressor” of those compounds (Fig. 2c, Table S2). To get a more expanded view of the metabolic changes, we lowered the FDR to 25%, which allowed us to detect besides tryptophan co-metabolites (5.5%), also differentially regulated acylcarnitines (25.5%) and lipids (20%) (Figures S2b, S2c, Table S2). Most of these metabolites were only modestly increased, but these data still give an impression of the direction of the metabolic rewiring in *SugctKO* kidney. 30% of detected lipids were prenols (arnamiol, armillarin, valtrate, isopetasoide, icariside B8), while the rest, where medium/long-chain fatty acids, one glycerophospholipid, and acyl choline (Table S2). Intriguingly, among the detected lipids were those associated with gut microflora metabolism, such as adipic acid [15], as well as those contributing to dysbiosis (palmitic acid, oleic acid, and linoleic acid) [16]. In addition, 24% of detected metabolites either did not have a eukaryotic origin and/or are co-regulated by gut bacteria (Fig. 2c, S2c, S3, Table S2, S3), indicating a prokaryotic contribution to the results that we observed. Considering that the kidney is held in a sterile environment, any presence of the non-host-derived metabolites was surprising. Interestingly, it has been shown that a substantial amount of the dietary tryptophan in the human gut is metabolized by bacteria [17]. In accordance with this, we detected in *SugctKO* mouse kidney increased indoleacrylic acid, which derives from indole-propionic acid [18], and is known as a suppressor of commensal inflammation [19]. It is important to keep in mind that gut bacteria-derived metabolites in kidney are pathological and linked to an early decline in renal function [20, 21] (Figure S3).

### 16S rRNA microbiome sequencing from stool DNA

**Fig. 3:**
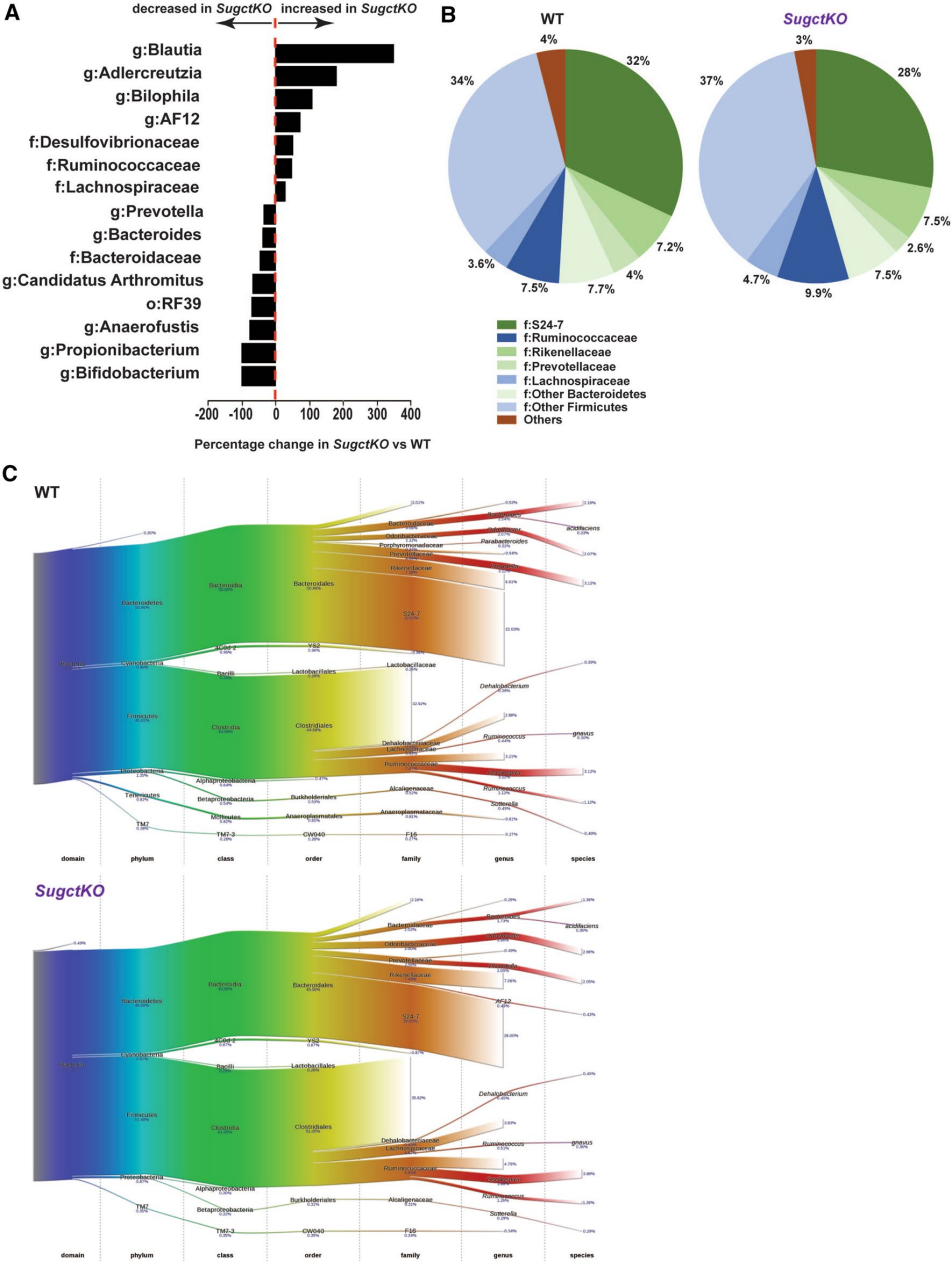
Diversity of gut bacterial microbiome in WT and *SugctKO* mice. **a** Graph depicting percentage change of indicated bacteria in *SugctKO* vs WT mice. The bars on the left and right side represent a percentage decrease and increase in *SugctKO* vs WT mice, respectively. Only significant differences between the groups with *p* < 0.1 are shown. **b** Pie chart representing changes in abundance of indicated families of bacteria. Only families with a change ≥ 10% are indicated. Shades of green indicate Bacteriodetes while shades of blue cover Firmicutes and red are others. **c** Sankey diagrams depicting the taxonomic flow from domain to species for WT and *SugctKO* mice

Following recent improved understanding of the relation between the gut microbiome and human health [22], we wanted to get an overview of the bacteria species in the gut of WT and *SugctKO* mice and performed 16S rRNA microbiome sequencing from stool DNA. We ensured exposure to the same microbial environment by co-housing both groups of mice and found substantial differences in the proportion and also type of bacteria species detected. In particular, there was a shift resulting in increase of firmicutes relative to bacteroidetes in *SugctKO* mice (Fig. 3a–c and Table S5). This is strongest in the Blautia genus containing the families Ruminococcaceae and Lachnospiraceae, which mirror the changes seen in a rat model of type 2 diabetes [23] as well as a metabolic syndrome in Mexican women with diabetes [24]. The metabolic effect of these bacterial families has been attributed to presence of carbohydrate-active enzymes, sugar transport, and metabolic pathways in their genome [25]. In addition, microbial diversity changes including the Blautia genus have also been linked to levels of indole-propionic acid [26] also identified in our metabolomics data. The second most increased genus, Adlercreutzia, has also been found increased in a diabetic rat model, and interestingly, this increase could be reverted by traditional Chinese medicine Xiexin Tang [23]. The third most increased genus in *SugctKO* mice is Bilophila, which is known to promote inflammation and aggravates high fat diet-induced metabolic dysfunctions in mice [27]. The fourth most increased genus AF12 has also been found increased in obese mice under high-fat diet [28]. At the same time, the common health-promoting gut microbes from the Bifidobacterium genus [29] were decreased (Fig. 3a–c).

Our results indicate that loss of *Sugct* affects the microbiome with changes closely resembling observed microbiome disbalance in metabolic diseases like diabetes.

### Antibiotic treatment reverses the metabolite profile of *SugctKO* mice

To investigate the potential contribution of intestinal bacteria to the metabolic changes detected in the *SugctKO* mouse kidney, we eliminated gut bacteria residing in the intestines by treatment with broad-spectrum antibiotics. The efficiency of antibiotic administration (hereinafter referred to as “abx”) on intestinal bacteria clearance was tested by 16S rRNA gene amplification of bacterial nucleic acids extracted from feces (Figure S4a, see also “Materials and methods”) [30]. We were not able to detect bacterial DNA after antibiotic treatment, indicating that the number of bacteria in the intestines of our mice was below the detection limit.

**Fig. 4:**
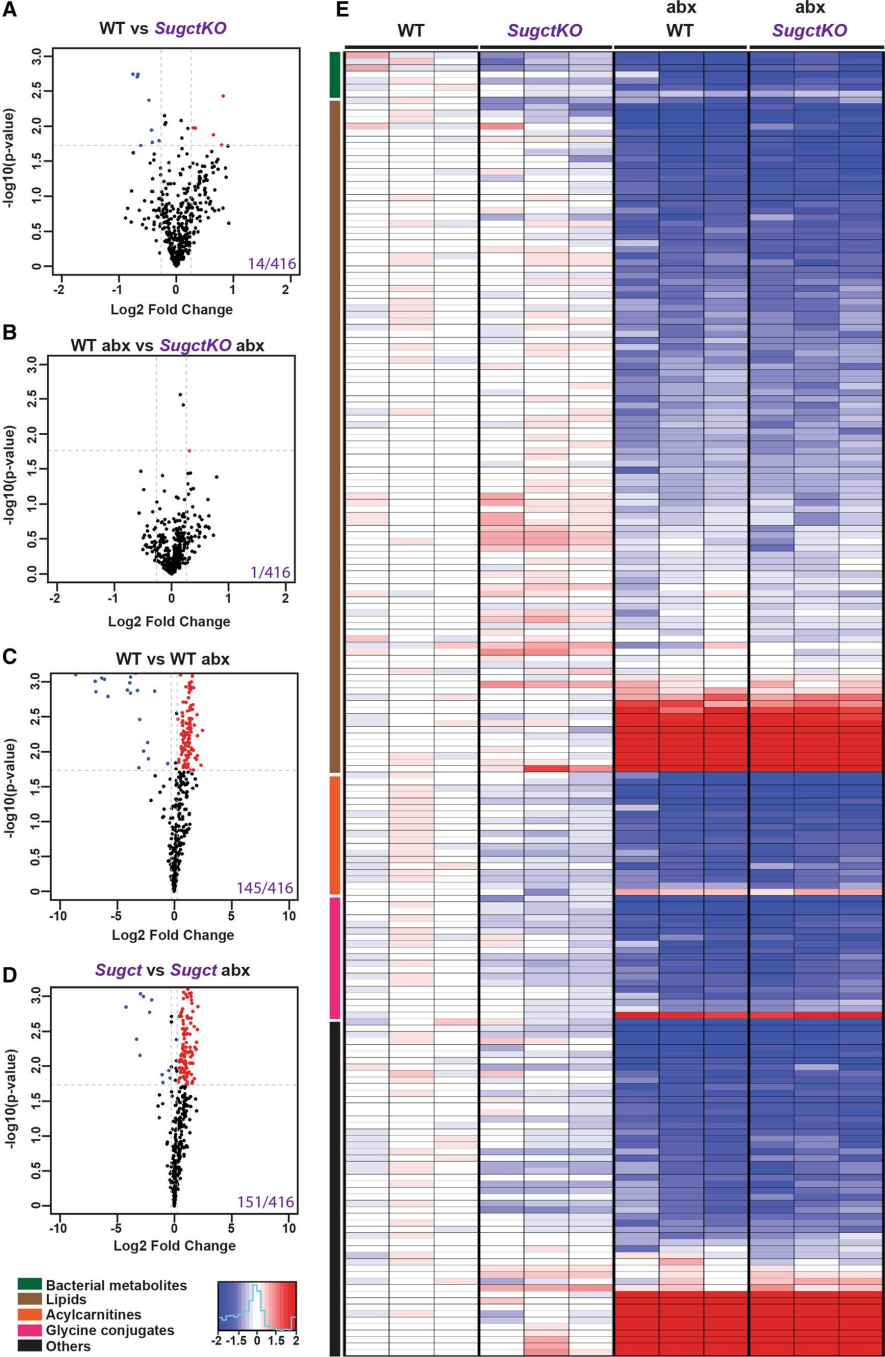
Contribution of gut microflora to plasma biochemistry in *SugctKO* mice. Untargeted LC–MS was performed from WT and *SugctKO* mouse plasma prior and post-antibiotic (abx) treatment. Three biological and three technical replicates were used for each tested condition. Volcano plots of 416 detected metabolites in mouse plasma from (**a**) WT vs *SugctKO*, **b** WT abx vs *SugctKO* abx, **c** WT vs WT abx, and **d** *SugctKO* vs *SugctKO* abx. Volcano plots were generated as previously described. *X*- and *y*-axis of depicted volcano plots display Log2FC and − log10*p* value, respectively. Significantly altered metabolites (*q* value = 0.05, *F*_c_ ≥ 1.2) are indicated by grey dashed lines and are colored in red and blue representing up- and downregulated metabolites, respectively. **e** Heatmap of the top 201 significantly altered (*q* value = 0.05, *F*_c_ ≥ 1.2) compounds from WT vs *SugctKO* mouse plasma, prior and post-antibiotic (abx) treatment. Red and blue color indicates up- and downregulation, respectively. Metabolites are clustered according to the following classes: bacteria-derived (green), lipids (brown), acylcarnitines (orange), glycine conjugates (pink), and others (black)

To compare WT and *SugctKO* mice prior and post-antibiotic administration, we collected blood from the same animal and the plasma was then subjected to metabolomic analysis by untargeted LC–MS. Using the same annotation criteria as described for the kidney (see “Materials and methods”), among 416 detected peaks (Fig. 4a–d, Table S4), we found that 145 and 151 metabolites were significantly changed after antibiotic treatment in WT and *SugctKO*, respectively (Fig. 4c, d). Furthermore, we tentatively annotated 14 compounds with significantly altered levels in WT vs *SugctKO* (Fig. 4a, 14/416), which were reduced to 1 metabolite after antibiotic treatment (Fig. 4b, 1/416), indicating that the differences in metabolites between WT and *SugctKO* were mostly governed by gut microbiota. Both control and mutant animals treated with antibiotics displayed very low levels of bacterial metabolites, further confirming the efficiency of the gut microflora clearance (Fig. 4e, green bar). In agreement with the LC–MS study in kidney (see Fig. 2), the alterations of metabolite expression in *SugctKO* mouse plasma, when compared to WT, included mainly lipids and acylcarnitines (Fig. 4e). Among the upregulated lipids (32%) were mostly lysophospholipids and phosphatidylserines (Fig. 4e, Table S4), which are known to accumulate in organs with high metabolic activity, as liver [31] and brain [32], but not in plasma. High levels of lysophospholipids in the bloodstream are linked to renal failure in hemodialysis [33], while the presence of phosphatidylserines is still not understood. Despite increased lipid levels, acylcarnitines appeared to be downregulated after antibiotic treatment (Fig. 4e, Table S4), suggesting that they may originate either directly or indirectly from microbiota. We also found significantly decreased concentrations of glycine-conjugated compounds, which serve as phase II metabolic products in chemical detoxification processes and are associated with the action of gut microflora [17] (Fig. 4e, pink bar). As expected, upon antibiotic administration, the levels of glycine conjugates became similarly low in WT abx and *SugctKO* abx plasma (pink bar, Fig. 4e, Table S4). Of interest, among other significant metabolites detected only in *SugctKO* mouse plasma (Fig. 4e, black bar) were dipeptides (Table S4). Although the consequences of dipeptides in plasma remain elusive, it is known that the clearance of not fully digested proteins depends on kidney and intestine functions [34]. In addition to metabolomic differences in *SugctKO* mouse plasma, we did not detect any morphological changes in kidney of young *SugctKO* mice prior and post abx treatment (Figure S4b). Nevertheless, we observed a mild increase of lipids in *SugctKO* mouse kidney that disappeared in gut microflora-deprived mice (Figure S4c, d), which agrees with the lipid imbalance in *SugctKO* mouse kidney (see Figs. 2c, 4e, S2, S3, Tables S2, S4).

Altogether, our data suggest that the antibiotic clearance of gut microflora in *SugctKO* mice alleviates alterations in the levels of lipids, acylcarnitines, bacterial metabolites, glycine conjugates, and dipeptides in *SugctKO* mouse plasma. This indicates that the absence of the gut microbiome restores the metabolic homeostasis in the animals harboring the *Sugct* mutation and may also suggest that the gut microbiome plays an important role in the GA3 disease.

### Age-associated obesity and glucose intolerance in *SugctKO* mice

**Fig. 5:**
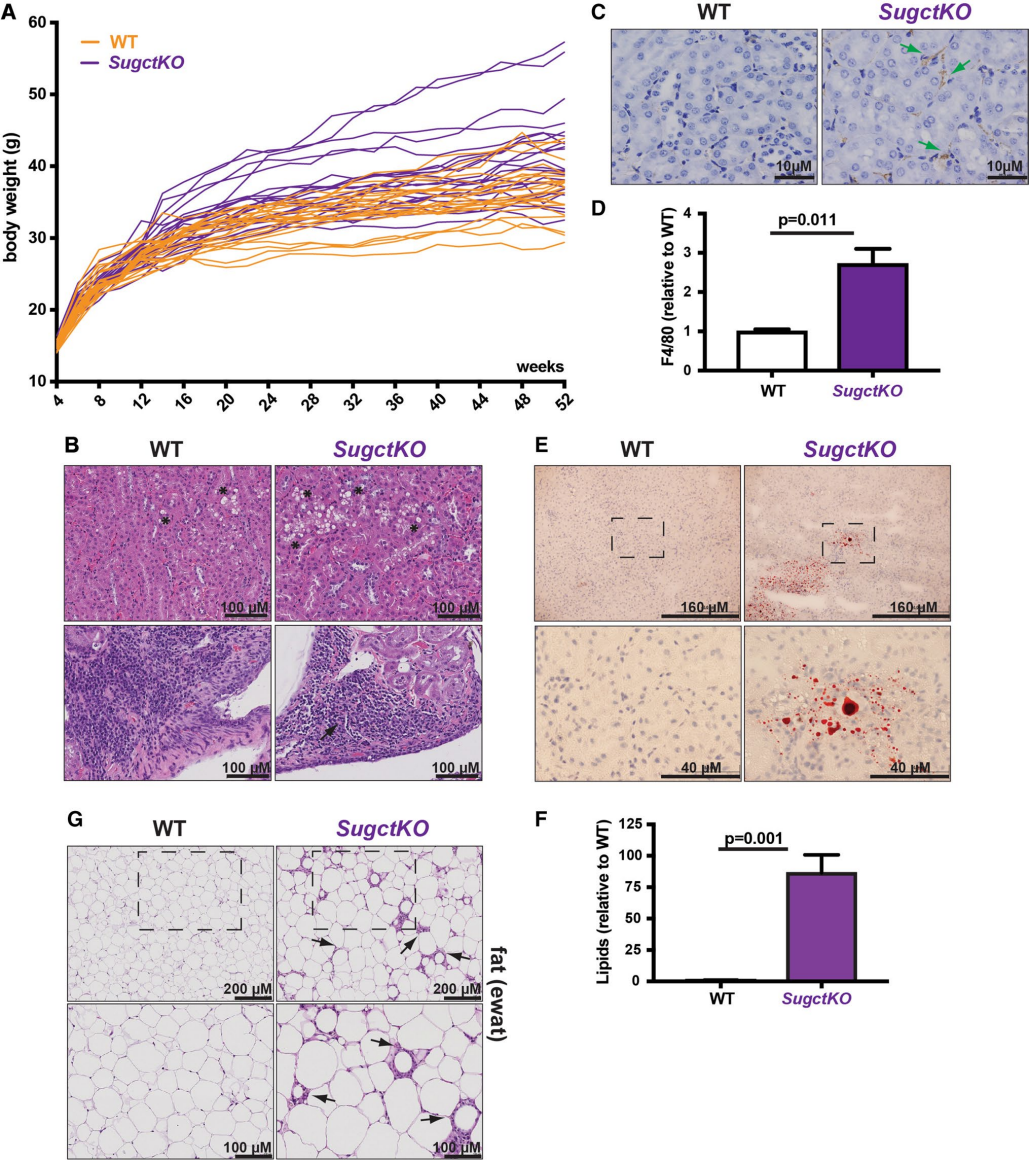
Ageing contributes to weight gain and kidney disease in *SugctKO* mice. **a** Body weight analysis of WT (*n* = 22, orange) and *SugctKO* (*n* = 22, purple) mice from 4 to 52 weeks after birth. Each line depicts the body weight of one experimental mouse. **b** Kidneys from 52-week-old WT (*n* = 10) and *SugctKO* (*n* = 10) mice were fixed and processed for H&E staining. Black asterisks indicate vacuolation, while black arrows indicate interstitial mononuclear cell infiltrate. **c** F4/80 immunostaining of paraffin-embedded kidney sections from 52-week-old WT (*n* = 3) and *SugctKO* (*n* = 3) mice. Green arrows indicate F4/80 positively stained macrophages. **d** Quantitative analysis of F4/80 positive staining from (**c**) using ImageJ. **e** ORO staining for lipids in the kidney from ~ 52-week-old WT (*n* = 4) and *SugctKO* (*n* = 4) mice. The region from the black-dashed square was 4X magnified on the picture below. **f** Quantification of ORO staining (**e**) relative to WT using ImageJ. **g** H&E staining of the fat (ewat) sections from ~ 52-week-old WT (*n* = 6) and *SugctKO* (*n* = 6) mice. Black arrows indicate adipocyte atrophy with granulomatous/mononuclear cell infiltrate (”crown-like structures”). The adipose tissue region from the black-dashed square was ×2 magnified in the picture below. Statistical analysis was done using two-tailed parametric paired *t* test

Initially, we did not observe obvious phenotypic changes in young *SugctKO* mice and their WT counterparts. However, renal lipidosis (see Fig. 2 and S2, S3, S4c, d) is associated with age-related progressive kidney failure and as a consequence, decreased physical and functional well-being of the patients [35, 36]. Based on this, we hypothesized that *Sugct* mutations could affect kidney functions in an age-dependent manner. Therefore, we aged WT and *SugctKO* animals for 52 weeks and monitored their body weight (Fig. 5a). Interestingly, the body weight of *SugctKO* mice was significantly elevated (≈ 41.5 g) when compared to their co-housed WT equivalents (≈ 36.7 g) at 52 weeks.

The significant increase of body weight in *SugctKO* mice could indicate metabolic dysfunction due to the loss of *Sugct*. Since SUGCT is highly expressed in kidney (see Figure S1e, f), where we observed metabolic changes in *SugctKO* mice (see Fig. 2, S2, S3), we collected kidneys from 52- to 58-week-old WT and mutant animals. We detected elevated number of vesicles in cytoplasm of renal tubular epithelial cells in WT mice [37], which in a subset of experimental *SugctKO* animals were further elevated (Fig. 5b, top panel). In addition, we noticed an increase of interstitial mononuclear cell infiltrate in *SugctKO* in comparison with WT mice (Fig. 5b, lower panel), an indication of inflammation. Therefore, we investigated the number of macrophages by tissue staining with F4/80 antibodies, best known as a marker of mature mouse macrophages and microglia [38] (Fig. 5c). We detected a threefold increased staining of macrophages in kidneys of aged *SugctKO* mice when compared to WT animals (Fig. 5d), which supports our hypothesis that metabolic changes in *SugctKO* mice may promote an inflammatory response.

Furthermore, there was lipid accumulation in the *SugctKO* kidney (Fig. 5e, f). Beside kidney, we analyzed histopathological changes in the liver (Figure S5a) and epididymal white adipose tissue (ewat; Fig. 5g, S5b) due to the observed weight gain in mutant animals (Fig. 5a). We observed micro- and macrovesicular steatosis in *SugctKO* mouse liver (Figure S5a) and we detected lipids by Oil Red O staining (Figure S5c, quantification shown in S5d). In addition, *SugctKO* mice displayed a greater degree of inflammation in adipose tissue, often forming “crown-like structures” [39] (Fig. 5g). This type of adipose tissue pathology indicates adipocyte death, which is often associated with macrophages surrounding dying adipocytes [39]. Despite the histopathological changes detected in kidney, liver, and epididymal white adipose tissue (ewat), no additional gross abnormalities were found in aged mutant animals.

In summary, we uncovered that ageing significantly contributes to the phenotype in *SugctKO* mice through increased body fat accumulation and progressive renal tubular vacuolation, which was accompanied with increased macrophage levels, fat accumulation in liver, and adipocyte death.

### Lysine-enriched diet aggravates histopathological changes in *SugctKO* mouse kidney

Diet composition has far-reaching effects on mammalian physiology [40]. Certain diet-induced pathologies that are severe in humans might only appear later or never in mice, since they are not exposed to varied diets. As previously shown in a mouse model for GA1, *GcdhKO* mice despite accumulating glutaric and 3-hydroxyglutaric acid, develop only mild motor deficits, unlike humans [41]. However, 4-week-old *GcdhKO* mice exposed to high-lysine diet display severe striatal degeneration typical of the human GA1 disease and 75% of the mice die within 3–12 days [42].

**Fig. 6:**
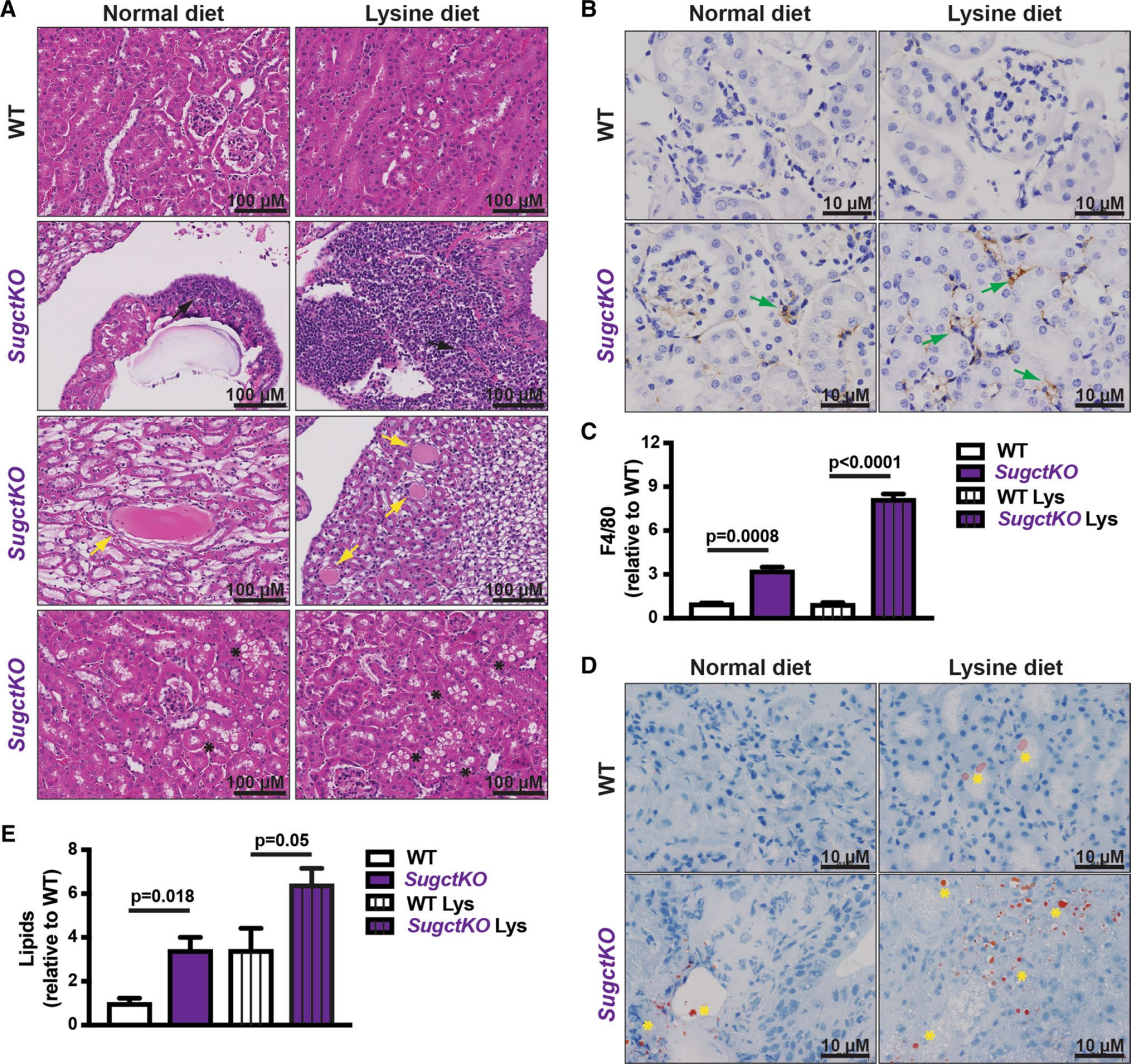
Dietary lysine supplementation aggravates the phenotype of *SugctKO* mice. 8–14 week-old mice were fed a normal [WT (*n* = 3), *SugctKO* (*n* = 4)] or 5xlysine diet [WT Lys (*n* = 4), and *SugctKO* Lys (*n* = 6)] for 20 weeks. **a** Kidneys were fixed and sections were subjected to H&E staining. Black arrows indicate mononuclear interstitial cell infiltrate, yellow arrows display tubule dilation, cystic change and tubular proteinosis, and black asterisks indicate macrovesicular renal tubule vacuolation, respectively. **b** F4/80 immunostaining of paraffin-embedded kidney sections from WT (*n* = 3) and *SugctKO* (*n* = 3) mice. Green arrows indicate F4/80 positively stained macrophages. **c** Quantification of (**b**) by ImageJ. **d** ORO staining of frozen kidney sections from WT (*n* = 3) and *SugctKO* (*n* = 3) mice to detect lipids. Yellow asterisks indicate lipid accumulation. **e** Quantification of (**d**) by Image J. Statistical analysis was done using two-tailed parametric paired *t* test

To accelerate and/or aggravate pathological changes observed in aged *SugctKO* mice (see Fig. 5b–f, S5), 8–12 week-old WT and *SugctKO* animals were fed with high-lysine diet for 20 weeks. The choice of diet was based on a previously reported study on *GcdhKO* mice fed with high-lysine or high-protein but not high-tryptophan diet [42] that substantially aggravated the phenotype. Although the histopathology of WT and *SugctKO* mice exposed to lysine diet (hereinafter referred to as “Lys”) revealed inflammation accompanied by steatosis in both WT Lys and *SugctKO* Lys mouse liver (data not shown), morphological changes in kidney were only detected in *SugctKO* Lys mice (Fig. 6a). *SugctKO* Lys developed acerbated interstitial mononuclear cell infiltrate, medullary tubule mineralization, tubular proteinosis, tubule dilation, and cystic change accompanied with increased renal tubular vacuolation compared to their corresponding controls (Fig. 6a). The presence of inflammatory cells in *SugctKO* Lys mouse kidney was confirmed by F4/80 staining (Fig. 6b, c). The number of detected macrophages in *SugctKO* on normal diet was threefold higher when compared to WT, while *SugctKO* Lys displayed almost a 2.5-fold increase in respect to *SugctKO* with no observed change in WT Lys mouse kidney (Fig. 6c). Moreover, the inflammatory state in kidney of *SugctKO* mice on normal diet was accompanied by an increase in lipid accumulation, which was aggravated by approximately threefold in *SugctKO* mouse kidney in relative comparison to WT and increased by additional twofold in *SugctKO* Lys (Fig. 6d, e). Interestingly, the lipid levels in WT Lys were similar to *SugctKO* on normal diet (Fig. 6d, e). Taken together, increased intake of dietary lysine in the context of *Sugct* deficiency elevates lipid accumulation and inflammatory cells in the kidney, suggesting that diet could also be a factor in the development of the GA3 disease.

## Discussion

We investigated the pathophysiology of the GA3 disease by performing metabolomics in kidney and plasma using our *SugctKO* mouse model. We found that *Sugct* loss of function is not only correlated with increased glutarate levels but also lipid/acylcarnitine imbalances and dysbiosis. In addition, we uncovered significant changes in the gut microbiome of *SugctKO* compared to WT mice. To further investigate the influence on the gut microflora metabolism caused by the *SugctKO* mutation, we expanded our study to mouse plasma, whose biochemistry is known to be strongly affected by bacterial metabolites that enter host’s circulatory system [17]. We found that the clearance of the gut microflora eliminated the metabolic differences between WT and *SugctKO* mouse plasma, indicating that the metabolism of *SugctKO* mice is regulated indirectly or directly by the microbiome. Furthermore, metabolic changes in *SugctKO* mice were connected to age-dependent susceptibility to excessive weight gain, as well as a potential decline in kidney function accompanied with pathological changes in liver and white adipose tissue. Last but not least, high-lysine diet was an important factor in aggravating and accelerating the severity of the observed pathologies in *SugctKO* mouse kidney. Overall, our results indicate that outcome of genetic mutations in *Sugct* is modulated by the microbiome, age, and diet.

**Fig. 7:**
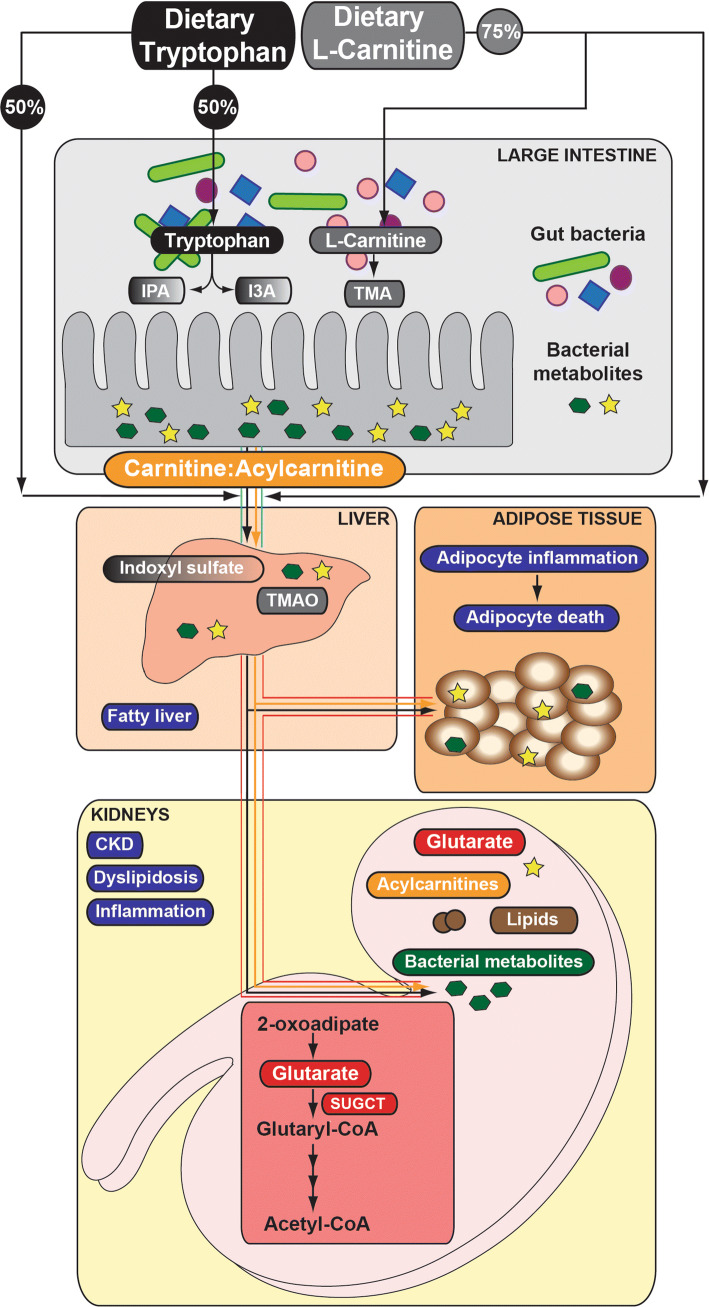
Generation, absorption, and circulation of gut bacteria-derived metabolites. Microbiome-derived metabolites are produced from dietary precursors, such as tryptophan and l-carnitine. Beneficial (IPA, I3A) and toxic [trimethylamine (TMA)] metabolites cross the intestinal epithelium to enter the liver through the portal vein (blue lines). Trimethylamine *N*-oxide (TMAO) and indoxyl sulfate are synthesized in the liver from TMA and indole, respectively. Once in circulation (red lines), toxic metabolites travel to distant tissues/organs/cells (adipose, kidney etc.). Accumulation of toxic solutes in the host contributes to numerous pathological changes and the development of disease

Phenotypic diversity of mammals is not only determined by the host, but also by its gut-residing microorganisms [43]. Since diet is the main source of metabolic precursors, biochemical compounds that are used in eukaryotic and prokaryotic signaling are either identical or very similar and sensing of metabolic cues depends on the communication between the host and microbes [22]. Despite that most amino acids are directly absorbed in the small intestine and further metabolized by the host [44, 45], the majority including tryptophan are degraded by the microbiota residing the large intestine [17, 46]. Such gut compartment-dependent metabolic preference depends on tryptophan’s beneficial influence on epithelial physiology and the mucosal immune system [47]. Apparently 40–60% of tryptophan is catabolized by tryptophanase-expressing bacteria to indole and its derivatives [17, 48] (Fig. 7), despite that eukaryotes retain the ability to metabolize tryptophan. Gut microbes possess different catalytic enzymes and often depend on mutual cooperation, thus any changes in tryptophan metabolism (including impaired endogenous tryptophan catabolism of the host) affects microbial homeostasis [49]. The shift between eukaryotic vs bacterial tryptophan metabolism can have far-reaching consequences for the host’s health, since indole-containing metabolites display either beneficial [indole-3-propionic acid (IPA) and indole-3-carboxaldehyde (I3A)] [50, 51] or toxic properties [indoxyl sulfate] [52]. Such harmful metabolites, if not cleared by the kidney and excreted in the urine, may circulate in the host’s body and accumulate in various organs, including kidney [52], liver, and/or adipose tissue leading to the development of severe pathologies over time (Fig. 7).

We have shown that loss of *Sugct* leads to modestly increased accumulation of a number of different acylcarnitines in the kidney (see Figure S2c, Table S2). Acylcarnitines are acyl esters of carnitine and essential compounds involved in energy production via fatty acids in mitochondria [53, 54]. l-carnitine (l-3-hydroxy-4-aminobutyrobetaine) is either synthesized in kidney and liver by the host from two essential amino acids, lysine and methionine (25%), or is directly absorbed from food (75%). Hypothetically, in the absence of functional SUGCT, the endogenous levels of carnitine in kidney might be higher due to its elevated precursor lysine, which cannot be efficiently catabolized to acetyl-CoA (see Fig. 1). The metabolic shift towards eukaryotic carnitine production increases the amount of unabsorbed dietary carnitine and triggers its bacterial metabolism into TMA (trimethylamine), whose oxidized form (trimethylamine N-oxide; TMAO) contributes to the fatty liver phenotype in the human population [55, 56]. Considering that the major source of renal ATP production is mitochondrial β-oxidation of free non-esterified fatty acids, carnitine imbalances could result in disturbances of the carnitine : acylcarnitine ratio and consequently leads to dyslipidosis followed by renal dysfunction [57, 58] (Fig. 7). Our discovery that loss of *Sugct* contributes to altered metabolic homeostasis between host and gut bacteria accompanied by dyslipidosis, is in line with a recent study of the GA3 disease, where a patient was reported with gastrointestinal disturbances [11]. Interestingly, treatment of the patient with antibiotics alleviated the symptoms, which indicates an important role of the gut microflora in the GA3 disease progression. Although this anecdotal finding supports our own results, a lot more GA3 patients will need to be treated with antibiotics before a final conclusion can be drawn.

There is growing evidence that beside environmental factors, genetic control shapes host-gut microbiota interactions [43, 59], which partially explains the emerging contribution of the microbiome to the onset of obesity [60, 61]. Until now, the GA3 disease has been poorly understood, underdiagnosed and in the majority of cases untreated. Therefore, mutations in *Sugct* should be considered as an important contributing factor in patients with metabolic disorders and detection of acylcarnitines/lipids/bacterial metabolites could serve as useful biomarkers for the choice of disease treatment.

## Materials and methods

All animal works were done in a humane way and were approved by Biological Resource Center (BRC) of Biopolis in A*STAR (IACUC #171268). Mice were housed under standard conditions, maintained on a 12-h light/dark cycle, and were co-housed. If not stated differently, mice were fed a standard chow diet containing 6% crude fat (Altromin, #1810) and were treated in compliance with the institutional guidelines for animal care and use. The high-lysine diet was prepared by adding free lysine to a standard diet (customized from Altromin) to achieve 4.7% total lysine (5X of the normal diet).

### Generation of knockout mice

Mouse genomic DNA harboring the *Sugct* locus was retrieved from the BAC clone RP23-451G10 (Invitrogen, PKB1129) and inserted into the pBlight-TK vector. *LoxP* recombination sites and Neo cassette were introduced flanking the third exon of murine *Sugct* genomic locus [62]. The targeting vector (PKB1318) was linearized by *Not*I digestion and electroporated into ES cells. Positive and negative selection with geneticin and ganciclovir, respectively, was followed by the homologous recombination screen of genomic DNA from ES cell colonies using Southern blot technique (5′ probe: chromosome 13, 17670059–17670559 bp; 3′ probe: chromosome 13, 17675399–17675899 bp). *Xho*I was a restriction enzyme used for screening both 5′ and 3′ end recombination events. Correctly targeted ES clones (9002, 9022, 9030) were used for the generation of the *Sugct* conditional knockout mouse strain. The *Sugct*^*flox*^ allele was obtained by crossing *Sugct* conditional knockout mice with β-actin-Flpe transgenic mice [63] [strain name: B6.Cg-Tg(ACTFLPe) 9205Dym/J; stock no.: 005703.; The Jackson Laboratory] to remove the Neo cassette. *Sugct*^*flox*^ mice were then crossed with β-actin-Cre transgenic mice [strain name: FVB/N-Tg(ACTB-cre)2Mrt/J; stock no.: 003376; The Jackson Laboratory] [64] to generate *Sugct*^+*/*null^ mice that were subsequently intercrossed to obtain *Sugct*^null/null^ mice and these were backcrossed at least 10 times to a C57BL/6 J background.

### RNA extraction and real-time PCR

RNA extraction was performed using the TRIzol^®^ Reagent (Ambion™ Life technologies, #15596018) extraction protocol. The TRIzol reagent was added to kidney and liver samples (~ 40 mg) in 2 mL lysing matrix D tubes (MP Biomedicals™, #6913-500). Tissues were homogenized in a Precellys 24 Dual^®^ (Bertin Technologies) at 4 °C using three cycles of 60 s at 5000 rpm. RNA concentration was measured with an eight-sample spectrophotometer (ND-800, NanoDrop^®^). cDNA was prepared using Maxima^®^ Reverse transcriptase (Fermentas Life Sciences, #EP0741), followed by qPCR using Maxima^®^ SYBR Green qPCR Master Mix (Fermentas Life Sciences, #K0251) with 10 ng of cDNA per reaction in a real-time thermal cycler (Corbett Research). Absolute quantification was obtained by a standard curve method using known concentration of serially diluted kidney RT-PCR product [65]. Primers used in this study are listed in Table S1.

### Western blot analysis

Frozen tissues were lysed in radioimmunoprecipitation assay (RIPA) buffer (50 mM Tris pH 8.0, 1% Nonidet P-40, 0.5% sodium deoxycholate, 0.1% SDS, 150 mM NaCl, 2X protease inhibitors [20 μg/mL each of leupeptin, chymostatin, and pepstatin; Chemicon, EI8, EI6, and EI10, respectively]) for 20 min on ice and clarified by centrifugation. Protein concentration was assessed using the BCA assay (Thermo Scientific, #23225). The protein extracts were separated by SDS-PAGE and transferred onto PDVF membrane (Millipore, IPVH0010) using a semi-dry trans-blot system. Buffer containing TBS with 0.1% Tween 20 (TBS-T) and 4% milk (Biorad, 1706404) was used as a blocking agent. The following primary antibodies were used: anti-C7ORF10 (Proteintech Group, #21589-1-AP) and anti-Hsp90 (BD Transduction Laboratories, #610419).

### LC–MS profiling from mouse kidney and plasma

Aqueous fractions were analyzed on a 6540 Quadrupole time-of-flight mass spectrometer (QTOF-MS, Agilent Technologies). The instrument was run in both positive and negative electrospray ionization (ESI) modes with scan range 50–1700 *m/z* and scan rate of 2 spectra/s. 5 μL of kidney and plasma extracts were injected onto Atlantis T3 Columns (100Å, 3 μm, 4.6 mm × 150 mm, #186003729). The mobile phases A and B consisted of 100% water with 0.1% formic acid and 100% acetonitrile with 0.1% formic acid, respectively. The gradient elution was as follows: 99.5% mobile phase A starting point held for 5 min, decreased over 15 min to 0% mobile phase A and held for 8 min. The column was re-equilibrated for 10 min. Total run time per sample was 38 min including equilibration.

### Kidney sample preparation for LC–MS analysis

Approximately 100 mg of kidney tissue per sample was homogenized twice with 800 µL of 1:1 methanol (MeOH):H_2_O and followed by centrifugation at 4 °C for 10 min. The supernatant was further partitioned twice using liquid–liquid extraction with 800 µL 4:1 dichloromethane:MeOH. The aqueous fraction was centrifuged, dried, resuspended in 200 µL 1:1 MeOH:H_2_O, and subsequently processed by LC–MS analysis.

### Antibiotic treatment, blood collection, and sample preparation for LC–MS analysis

Streptomycin sulfate salt (Sigma-Aldrich, #S6501) and penicillin G sodium salt (Sigma-Aldrich, #P3032) were added to drinking water at the concentration of 2 g/L and 1500 U/mL, respectively. The drinking water was changed every 3 days during the 4-week long treatment. Blood from WT and *SugctKO* mice (5-week-old prior and 9-week-old post-treatment) was collected into lithium/heparin-coated vacutainers (Microvette 500 LH, Sarstedt, #20.1345.100) either by submandibular bleeding or by terminal cardiac puncture. Vacutainers were centrifuged for 5 min at 3000 rpm at 4 °C and supernatant was immediately frozen at − 80 °C. 200 µL of acetonitrile was added to 50 µL of blood plasma per sample. The mixture was vortexed vigorously for 1 min and incubated on ice for 10 min. Subsequently, the mixture was centrifuged at 14,000*g* for 15 min and the supernatant was transferred for LC–MS analysis. Blank and pooled quality control (QC) samples were prepared for instrument QC purposes.

### LC–MS data and statistical analysis

The MassHunter Profinder software (Agilent technologies, version 6.0) was used for data extraction with the following parameters: batch recursive feature extraction; ion species allowed: H^+^, Na^+^, and K^+^; charge states were limited to a maximum of 2; compound ion count threshold was set to 2 or more ions; Extracted Ion Chromatogram (EIC) tolerance for mass was set to 10 ppm; retention time 2–13 min; and absolute height > 1000 counts. Peak area values from precursor ion chromatograms, extracted by the Mass Profiler Professional (MPP) software, were averaged over technical triplicate experiments by geometric mean. Compound identities for extracted precursor ion peaks were assigned by two modes of identification. First, identifications by isotopic standards were considered identified with full confidence. Second, the remainder of peaks without standard-based verification was queried against a composite database of formula-mass information from the NIST version 14 [66], HMDB version 3 [67], MassBank [68], and LIPIDBLAST [69] libraries based on the similarity of neutral mass (mass error). The distribution of mass errors against the best matching compounds was decomposed into true and false identifications, where the sub-distribution of the false identifications was learned from that of the second-best matching database entries of all peaks. The mixture deconvolution enabled us to compute the posterior probability of true identification, up to unique formula (subject to the uniqueness of mass value per structure). Compound assignments of probability 0.8 or above were taken as positive identifications, which corresponded to less than 20 ppm mass error in all cases. Finally, the identifications resulting in assignment of two or more isomers (of identical formula) were further removed from the data to avoid ambiguity of identification. Positive and negative ion mode data were processed separately throughout this process. For statistical analysis, the data were log-transformed (base 2) after adding a small fudge factor, determined by the 10 percentile point of each data set (by organ, by ionization mode) to avoid over-estimated fold change originating from noisy peaks with low peak area values. All statistical analyses were performed using R (http://cran.r-project.org), including principal component analysis, two-sample independent *t* tests and *q* value calculation, and generation of heat maps and volcano plots. In differential abundance analysis, the quantitative data matrix was obtained by summing peak area values of all peaks with identical compound identity in each sample, since abundant species tend to appear in multiple peaks, indicating long chromatographic elution. Two-sample *t* test followed by multiple testing correction (*q* value, [70]) was used for testing differential abundance in all comparisons. For the kidney data, as the total number of significant findings was very small, we applied *q* value of 0.15 (15% FDR) as the threshold of statistical significance. In the plasma data, we applied *q* value of 0.05 (5% FDR) to control the total number of false discoveries reasonably low. We also applied minimal 20% change as additional requirement in the selection of significant findings.

### Bacterial DNA extraction from mouse fecal pellets and 16S rRNA gene PCR amplification

Feces were freshly collected and immediately snap frozen in liquid nitrogen before extraction using QIAamp Fast DNA Stool Mini Kit (#51604). Extracted bacterial DNA was amplified using the primer pair 338F* (PKO6597) and 1061R (PKO6598), as has been described [30]. All primers are listed in Table S1.

### 16S rRNA gene amplification and sequencing

We used the method that has been described by Ong et al. [30] and recently by Ta et al. [71]. Our comparison groups consisted of 7 WT and 7 *SugctKO* mice that were all co-housed for consistent exposure to the same microbial environment, which meant that always 2 WT and 2 *SugctKO* mice were housed in the same cage. We identified significant differences between the averages of the two groups with one-sided *t* test assuming equal variance (*p* < 0.1) and rank them by percent increase or decrease in *SugctKO* mice (Fig. 3a). In addition, we summarize the total composition of the microbiome as pie charts (Fig. 3b) and Sankey diagrams (Fig. 3c) generated using a sequence similarity cut off [30, 71] and the bacterial accession in the Greengenes database.

### Histology

For hematoxylin and eosin (H&E) staining, tissues were fixed in 10% neutral buffered formalin (NBF, Sigma-Aldrich, HT501128) for 18–24 h, transferred to ice–cold 70% ethanol, and embedded in paraffin blocks followed by the staining of tissues sections.

### Oil red O (ORO) staining

Slides with frozen tissue sections were dipped in isopropanol 60% for 5 min and stained in filtered ORO working solution [3:2—ORO stock (1% ORO in isopropanol): Dextrin (1% Dextrin in water)], followed by rinse in isopropanol 60%. Then, slides were counterstained in hematoxylin solution for 2 min, washed in deionized water for 1 min, followed by the wash in tap water for 5 min.

### F4/80 immunohistochemistry

Slides with paraffin-embedded tissue sections were deparaffinized and rehydrated at room temperature for 5 min, followed by blocking of endogenous peroxidase in methanol/H_2_O_2_ (1:33.3) for 15 min and rehydration in water for 1 min. Subsequently, slides were incubated with 20 mg/mL Proteinase K (Invitrogen, #25530-049) for 20 min at 37 °C, washed 2× in Phosphate Buffered Saline (PBS), and blocked with 1% Bovine Serum Albumin (BSA, Sigma-Aldrich, #A7906-100G) for 45 min at RT. After blocking, slides were incubated with primary rat anti-mouse F4/80 (Serotec, #MCA497G) antibodies (1/200) at RT, washed 3× in PBS followed by incubation with AffiniPure rabbit anti-rat secondary antibodies (1:5000, Jackson ImmunoResearch, #312 005-003) for 1 h at RT. Slides were washed 3X in PBS, treated for 30 min with Dako Envision + System-HRP Labeled Polymer Anti-mouse (DAKO, #K4001) for 30 min at RT, washed 3× in PBS, incubated with Dako Liquid 3,3′-Diaminobenzidine (DAB) + Substrate Chromogen System [(DAKO, #K3468), 1 drop of the DAB Chromogen per mL of Substrate Buffer] for 5 min at RT, counterstained with hematoxylin for 5 min at RT, washed 3X in PBS, rinsed with running water, and mounted.

### Statistics

All experiments were performed with a minimum of three animals. For data that followed a normal distribution, statistical significance was tested using the two-way Student *t* test. For LC–MS plasma analysis, we used two-way ANOVA. Data were represented, as the mean value and error bars represent the standard error of the mean (SEM). *p* value was calculated with two-tailed paired *t* test with 95% level of confidence.

## Electronic supplementary material

Below is the link to the electronic supplementary material.

Supplementary material 1 Generation of *Sugct* knockout mice. (A) *Sugct* targeting strategy: lane I—*Sugct* genomic locus, lane II—gene targeting construct, lane III—*Sugct* flox locus after Frt-mediated recombination (*Sugct*^*flox*^ allele), lane IV—*Sugct* null locus after Cre-mediated recombination. Coding exons are depicted as black boxes and are numbered above. The neomycin selectable marker (Neo), LoxP, and FRT sequences are shown as a green box, grey triangles, and red rectangles, respectively. Restriction enzyme recognition sites are shown with black vertical arrows: *Xho*I. Horizontal arrows denote PCR primers used for genotyping animals (P1, P2, P3). (B) Southern blot analysis of genomic DNA extracted from WT and *Sugct*^+*/flox*^ embryonic stem cell clones. Genomic DNA was digested with *Xho*I and hybridized with a 5′ or a 3′ probe. (C) Genotyping PCR of DNA extracted from tails of WT, *Sugct*^+*/flox*^ and *Sugct*^+*/null*^ mice using primers P1, P2, and P3. (D) Expected and observed frequency (%) of P21 pups (200 pups counted in total) obtained from heterozygous *Sugct* intercrosses. (E) Real-time (R-T) PCR of *Sugct* mRNA expression levels in liver, kidney, lungs, brain, skin, muscle, heart, spleen, stomach, and testis. Sequences of primers used are listed in Table S1. There was no signal detected in *SugctKO* organs (data not shown). (F) Western blot analysis of SUGCT expression in liver, kidney, lungs, brain, and spleen from WT and *SugctKO* (P60) mice. Results represent three independent experiments. HSP90 served as a loading control (JPEG 2776 kb)

Supplementary material 2 Related to Fig. 2. LC–MS-based metabolomic profiling of kidney extracts from WT and *SugctKO* mice. (A) Graphs representing fold change (Fc) and significance (p) of detected glutarylcarnitine and acetylcarnitine from WT (white) and *SugctKO* (purple) mouse kidney. (B) Volcano plot of 808 metabolites detected in WT vs *SugctKO* mouse kidney. The volcano plot was generated as a log scaled axes of fold change (Log2, x-axis) and *p* value (-log10, y-axis). Significantly altered metabolites (*p* value ≤ 0.05, Fc ≥ 1.2) are indicated by dashed grey lines and colored in red and blue representing up- and downregulated metabolites, respectively. (C) Heatmap depicting up- (red) and downregulated (blue) compounds (*p* value ≤ 0.05, Fc ≥ 1.2) from WT vs *SugctKO* mouse kidney. Metabolites are clustered according to the following classes: tryptophan metabolism (yellow), acylcarnitines (orange), lipids (brown), metabolites of bacterial origin (green), and others (black). The estimated false discovery rate at *p* value cutoff 0.05 was 25.4% (*q* value = 0.254) (JPEG 3413 kb)

Supplementary material 3 Related to Fig. 2. Accumulation of the microbiome-derived metabolites in the kidney. Various metabolites are generated solely in the presence of the gut microflora, enter the blood flow, and possibly accumulate in kidney. The graphs depict fold change (Fc) differences of significantly altered (*q* value = 0.15, Fc ≥ 1.2) bacteria-derived molecules (p) between WT (white) and *SugctKO* mouse kidney (purple) (JPEG 1684 kb)

Supplementary material 4 Related to Fig. 4. Effects of antibiotic treatment on the plasma metabolome in *SugctKO* mice. (A) Bacterial DNA extraction from feces and subsequent 16S rRNA gene PCR amplification in WT (n = 3) and *SugctKO* (n = 3) mice prior and post-antibiotic (abx) treatment. (B) H&E staining of kidney sections from 12-week-old WT (n = 3) and *SugctKO* (n = 3) mice without treatment and treated for 4 weeks with abx. (C) ORO staining of frozen kidney sections from (B). Black arrow indicates lipid accumulation. (D) Quantification of ORO staining using ImageJ. Statistical analysis was done using two-tailed parametric paired *t* test (JPEG 4723 kb)

Supplementary material 5 Related to Fig. 5. Increased hepatic and subcutaneous fat accumulation in aged *SugctKO* mice. (A) H&E staining of the liver from ~ 52-week-old WT (n = 6) and *SugctKO* (n = 6) mice. Yellow arrows indicate hepatocellular macrovesicular lipids accumulation. (B) Body, liver, fat (ewat), and kidney (left and right) were weighed in 52-week-old WT (n = 6, white) and *SugctKO* (n = 6, purple) mice. (C) ORO staining of the liver from ~ 52-week-old WT (n = 4) and *SugctKO* (n = 4) mice. The region from the black-dashed square was 4X magnified on the picture below. (D) Quantification of ORO staining (C) relative to WT using ImageJ. Statistical analysis was done using two-tailed parametric paired *t* test (JPEG 5356 kb)

Supplementary material 6 Related to Figs. S1 and S4. Primers used in this manuscript (XLSX 9 kb)

Supplementary material 7 Related to Figs. 2 and S2. The combined list of detected metabolites from WT and *SugctKO* mouse kidney (XLSX 792 kb)

Supplementary material 8 Related to Figs. 2, 4, S2, and S3. The list of chemical standards used in our metabolomic study (XLSX 10 kb)

Supplementary material 9 Related to Fig. 4. The combined list of detected metabolites from WT vs *SugctKO*, WT abx vs *SugctKO* abx, WT vs WT abx, and *SugctKO* vs *SugctKO* abx mouse plasma (XLSX 573 kb)

Supplementary material 10 Related to Fig. 3. 16S sequencing of the microbiome (XLSX 44 kb)
