# Supplementary figures and images for: Knockout of the non-essential gene SUGCT creates diet-linked, age-related microbiome disbalance with a diabetes-like metabolic syndrome phenotype
Source: Cell Mol Life Sci. 2019 Nov 13;77(17):3423–39. doi: 10.1007/s00018-019-03359-z (PMC7426296; doi:10.1007/s00018-019-03359-z)

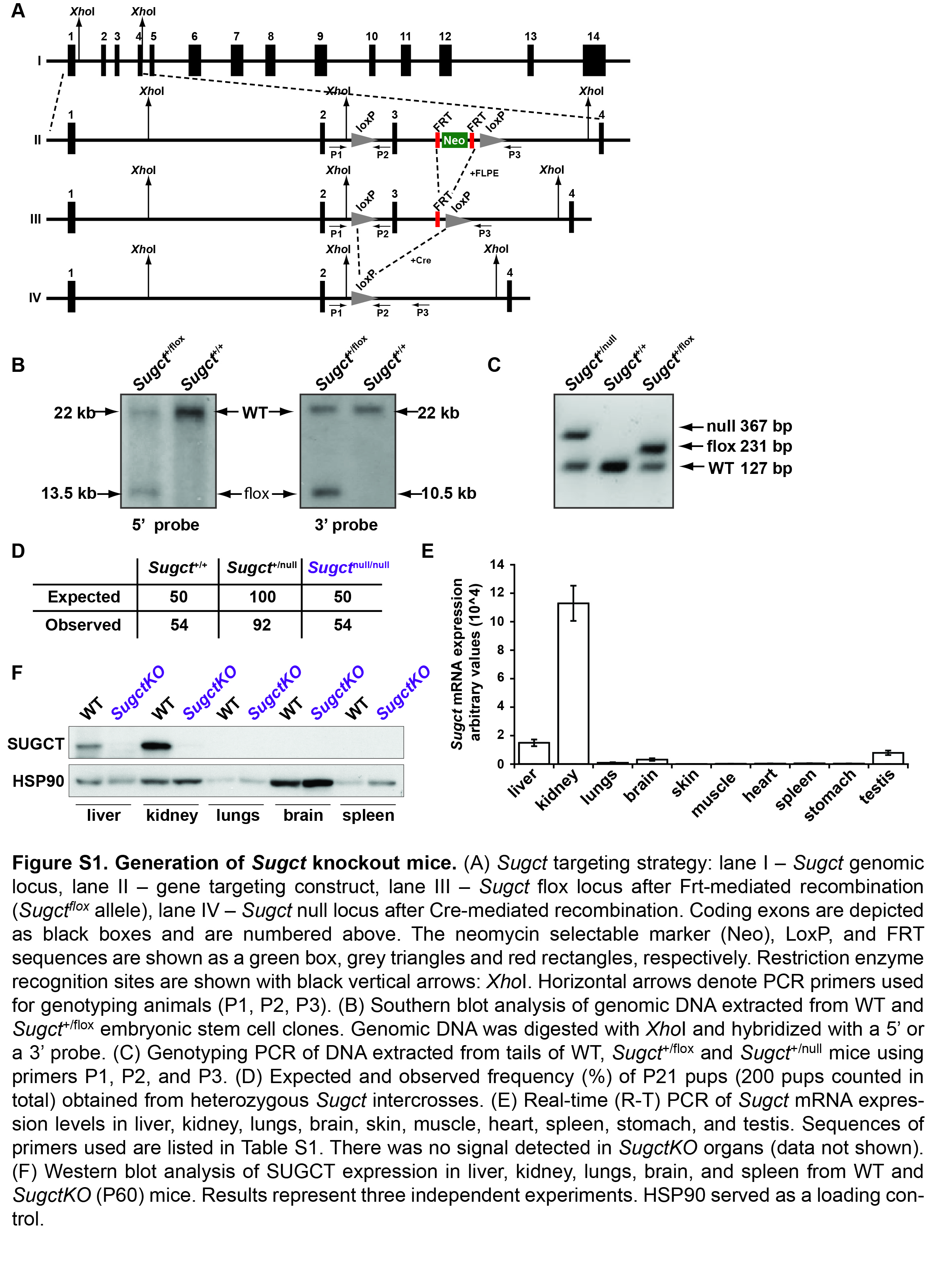

Supplement: Supplementary file 1 — Supplementary material 1 Generation of Sugct knockout mice. (A) Sugct targeting strategy: lane I—Sugct genomic locus, lane II—gene targeting construct, lane III—Sugct flox locus after Frt-mediated recombination (Sugctflox allele), lane IV—Sugct null locus after Cre-mediated recombination. Coding exons are depicted as black boxes and are numbered above. The neomycin selectable marker (Neo), LoxP, and FRT sequences are shown as a green box, grey triangles, and red rectangles, respectively. Restriction enzyme recognition sites are shown with black vertical arrows: XhoI. Horizontal arrows denote PCR primers used for genotyping animals (P1, P2, P3). (B) Southern blot analysis of genomic DNA extracted from WT and Sugct+/flox embryonic stem cell clones. Genomic DNA was digested with XhoI and hybridized with a 5′ or a 3′ probe. (C) Genotyping PCR of DNA extracted from tails of WT, Sugct+/flox and Sugct+/null mice using primers P1, P2, and P3. (D) Expected and observed frequency (%) of P21 pups (200 pups counted in total) obtained from heterozygous Sugct intercrosses. (E) Real-time (R-T) PCR of Sugct mRNA expression levels in liver, kidney, lungs, brain, skin, muscle, heart, spleen, stomach, and testis. Sequences of primers used are listed in Table S1. There was no signal detected in SugctKO organs (data not shown). (F) Western blot analysis of SUGCT expression in liver, kidney, lungs, brain, and spleen from WT and SugctKO (P60) mice. Results represent three independent experiments. HSP90 served as a loading control (JPEG 2776 kb) [file 18_2019_3359_MOESM1_ESM.jpg]

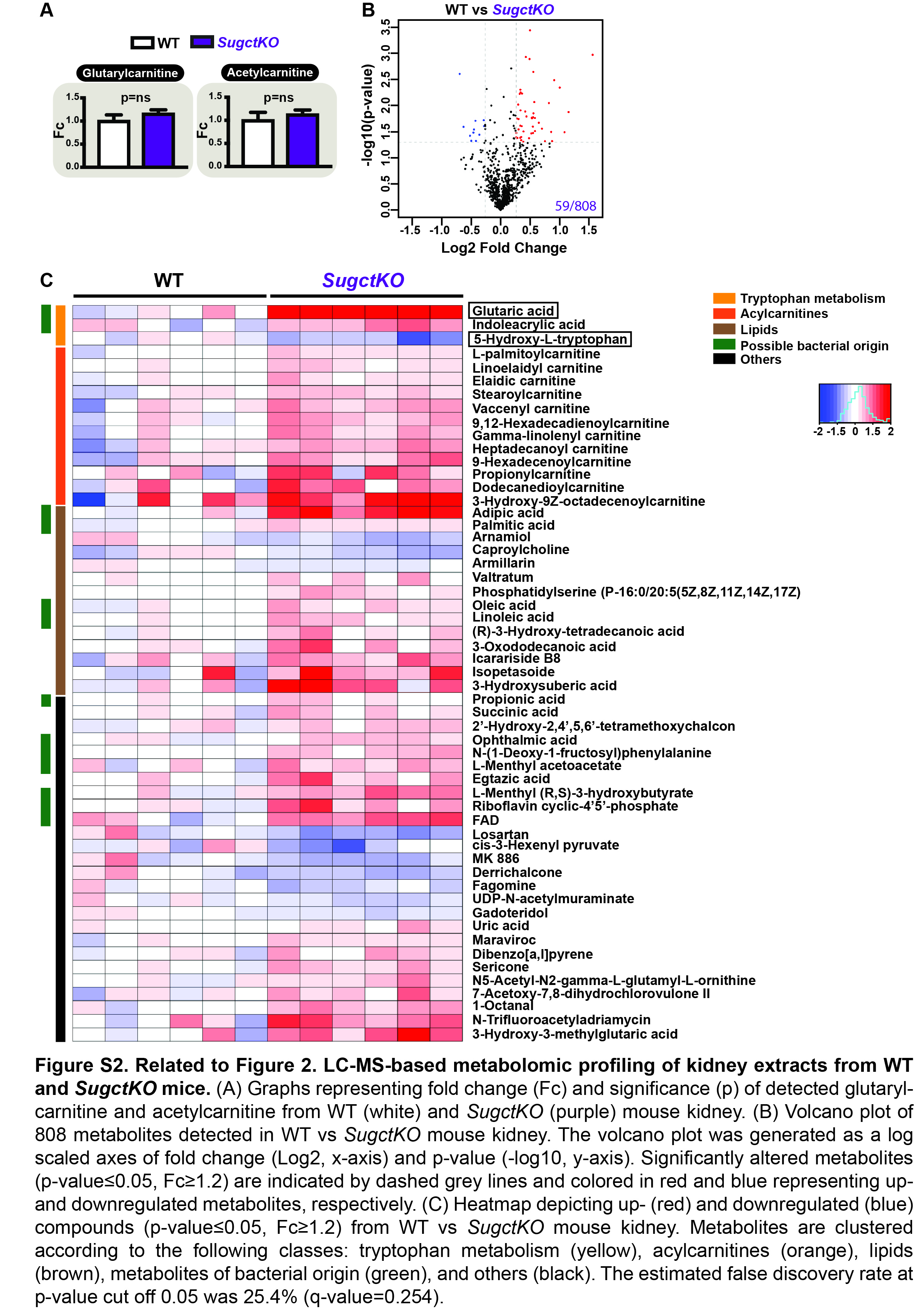

Supplement: Supplementary file 2 — Supplementary material 2 Related to Fig. 2. LC–MS-based metabolomic profiling of kidney extracts from WT and SugctKO mice. (A) Graphs representing fold change (Fc) and significance (p) of detected glutarylcarnitine and acetylcarnitine from WT (white) and SugctKO (purple) mouse kidney. (B) Volcano plot of 808 metabolites detected in WT vs SugctKO mouse kidney. The volcano plot was generated as a log scaled axes of fold change (Log2, x-axis) and p value (-log10, y-axis). Significantly altered metabolites (p value ≤ 0.05, Fc ≥ 1.2) are indicated by dashed grey lines and colored in red and blue representing up- and downregulated metabolites, respectively. (C) Heatmap depicting up- (red) and downregulated (blue) compounds (p value ≤ 0.05, Fc ≥ 1.2) from WT vs SugctKO mouse kidney. Metabolites are clustered according to the following classes: tryptophan metabolism (yellow), acylcarnitines (orange), lipids (brown), metabolites of bacterial origin (green), and others (black). The estimated false discovery rate at p value cutoff 0.05 was 25.4% (q value = 0.254) (JPEG 3413 kb) [file 18_2019_3359_MOESM2_ESM.jpg]

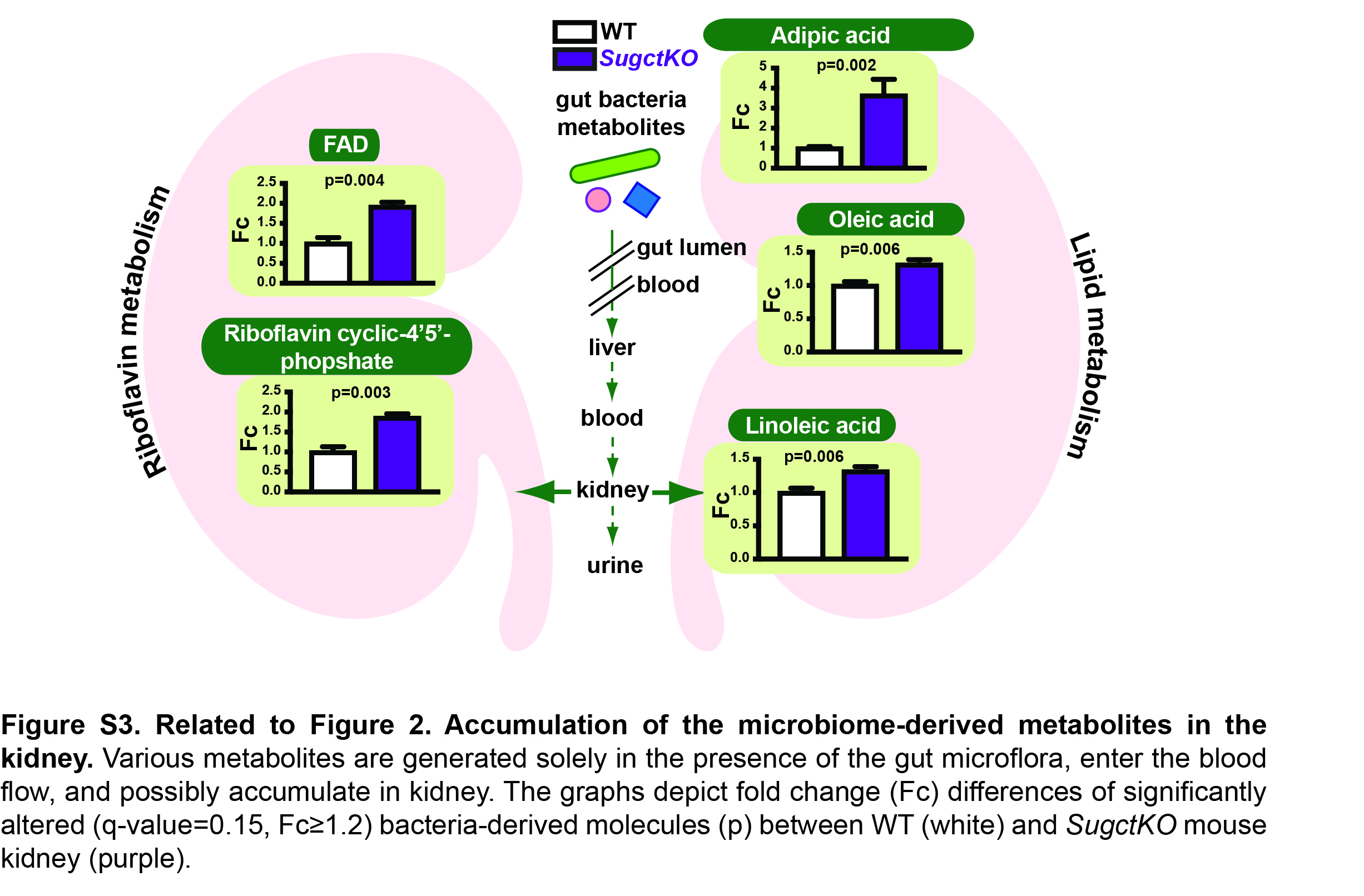

Supplement: Supplementary file 3 — Supplementary material 3 Related to Fig. 2. Accumulation of the microbiome-derived metabolites in the kidney. Various metabolites are generated solely in the presence of the gut microflora, enter the blood flow, and possibly accumulate in kidney. The graphs depict fold change (Fc) differences of significantly altered (q value = 0.15, Fc ≥ 1.2) bacteria-derived molecules (p) between WT (white) and SugctKO mouse kidney (purple) (JPEG 1684 kb) [file 18_2019_3359_MOESM3_ESM.jpg]

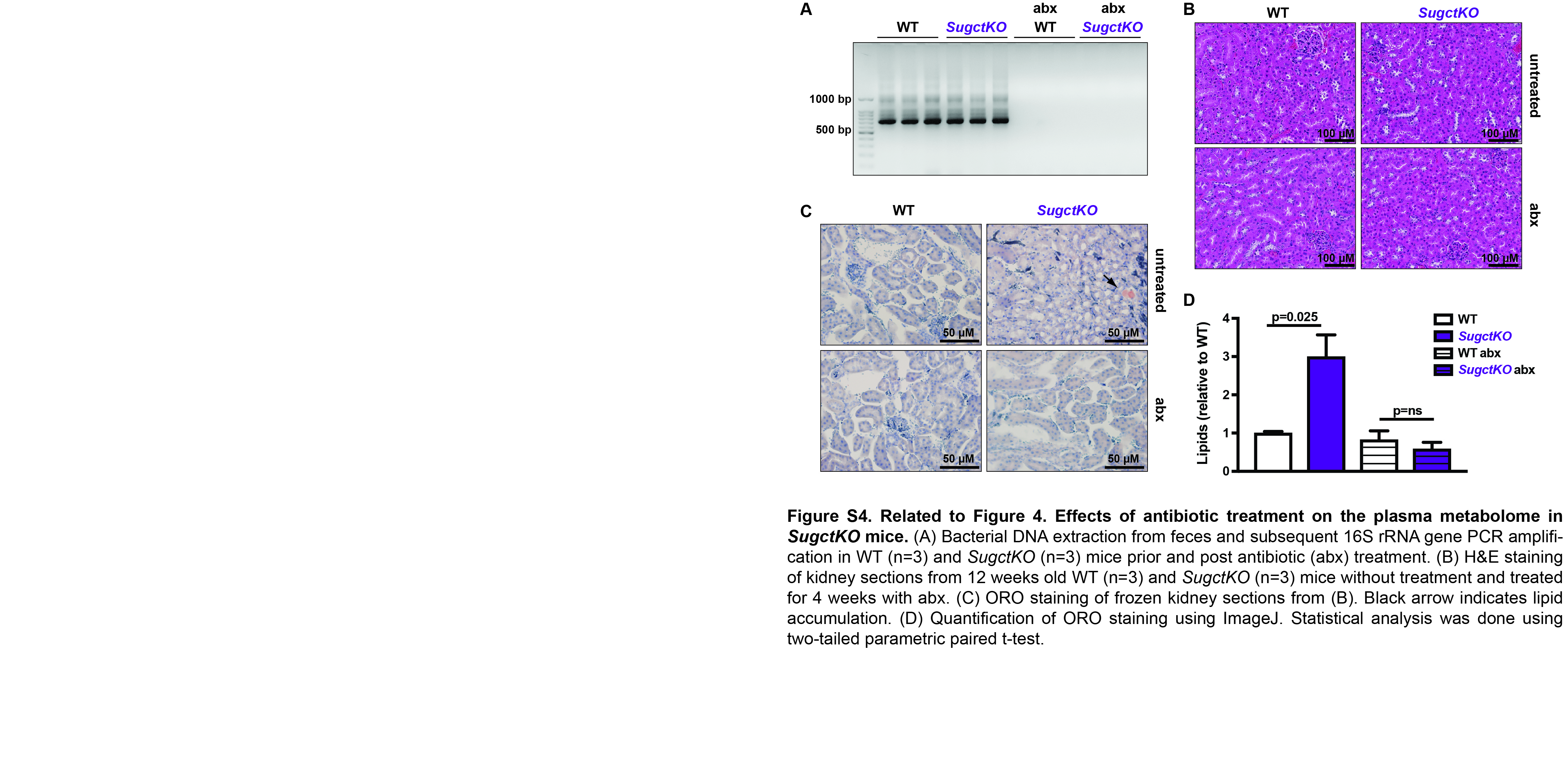

Supplement: Supplementary file 4 — Supplementary material 4 Related to Fig. 4. Effects of antibiotic treatment on the plasma metabolome in SugctKO mice. (A) Bacterial DNA extraction from feces and subsequent 16S rRNA gene PCR amplification in WT (n = 3) and SugctKO (n = 3) mice prior and post-antibiotic (abx) treatment. (B) H&E staining of kidney sections from 12-week-old WT (n = 3) and SugctKO (n = 3) mice without treatment and treated for 4 weeks with abx. (C) ORO staining of frozen kidney sections from (B). Black arrow indicates lipid accumulation. (D) Quantification of ORO staining using ImageJ. Statistical analysis was done using two-tailed parametric paired t test (JPEG 4723 kb) [file 18_2019_3359_MOESM4_ESM.jpg]

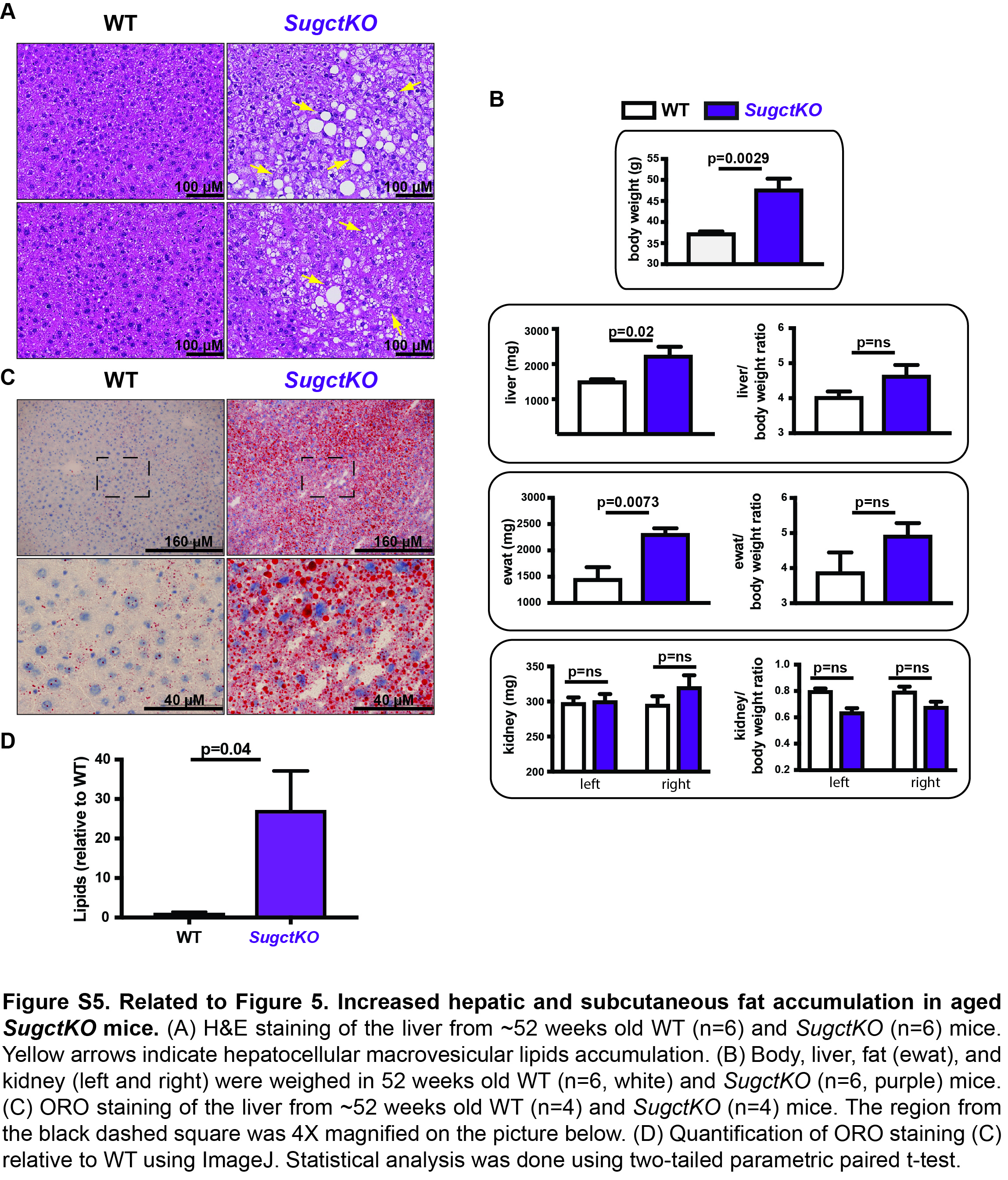

Supplement: Supplementary file 5 — Supplementary material 5 Related to Fig. 5. Increased hepatic and subcutaneous fat accumulation in aged SugctKO mice. (A) H&E staining of the liver from ~ 52-week-old WT (n = 6) and SugctKO (n = 6) mice. Yellow arrows indicate hepatocellular macrovesicular lipids accumulation. (B) Body, liver, fat (ewat), and kidney (left and right) were weighed in 52-week-old WT (n = 6, white) and SugctKO (n = 6, purple) mice. (C) ORO staining of the liver from ~ 52-week-old WT (n = 4) and SugctKO (n = 4) mice. The region from the black-dashed square was 4X magnified on the picture below. (D) Quantification of ORO staining (C) relative to WT using ImageJ. Statistical analysis was done using two-tailed parametric paired t test (JPEG 5356 kb) [file 18_2019_3359_MOESM5_ESM.jpg]
